# The Chinese version of patient-doctor-relationship questionnaire (PDRQ-9): Factor structure, validation, and IRT psychometric analysis

**DOI:** 10.3389/fpsyt.2023.1117174

**Published:** 2023-02-16

**Authors:** Yufei Wang, Aoxue Wu, Yinan Jiang, Yanping Duan, Wenqi Geng, Lin Wan, Jiarui Li, Jianhua Du, Jiaojiao Hu, Jing Jiang, Lili Shi, Jing Wei

**Affiliations:** ^1^Department of Psychological Medicine, Peking Union Medical College Hospital, Chinese Academy of Medical Sciences and Peking Union Medical College, Beijing, China; ^2^4+4 Medical Doctor Program, Chinese Academy of Medical Sciences and Peking Union Medical College, Beijing, China; ^3^Eight-Year Medical Doctor Program, Chinese Academy of Medical Sciences and Peking Union Medical College, Beijing, China

**Keywords:** doctor-patient relationship, patient safety, item response theory, PDRQ, factor analysis

## Abstract

**Objective:**

The patient-doctor relationship has been considered as a crucial concept in primary healthcare, while the medical reform launched by the Chinese government in 2009 has brought significant changes to the healthcare system, which made it urgent to introduce reliable measurement instruments for assessing today’s doctor-patient relationship in China. This study examined the psychometric properties of the Chinese version of the Patient-Doctor-Relationship Questionnaire-9 item (PDRQ-9) scale among general hospital inpatients in China.

**Materials and methods:**

A total of 203 participants responded to the survey, of which 39 completed retest after 7 days. Factor analyses were used to test the construct validity of the scale. Convergent validity was evaluated by the correlation between PDRQ-9 and depressive symptoms measured using PHQ-9 (Patient Health Questionnaire Depression Scale-9 item). Both multidimensional item response theory (MIRT) and unidimensional item response theory (IRT) framework were used to estimate the parameters of each item.

**Results:**

The two-factor model of relationship quality and treatment quality was supported (χ^2^/*df* = 1.494, GFI = 0.925, RMSEA = 0.071, RMR = 0.008, CFI = 0.985, NFI = 0.958, NNFI = 0.980, TLI = 0.980, IFI = 0.986). The PDRQ-9 and both subscales showed significant correlation with PHQ-9 (*r* = −0.196∼−0.309) and good internal consistency (Cronbach’s alpha = 0.865∼0.933). ANCOVA analysis adjusted with age revealed significant difference in PDRQ-9 ratings between patients with or without significant depressive symptoms (*P* = 0.019). The 7-day test-retest reliability of the scale was 0.730. The MIRT model of full scale and IRT models of both subscales showed high discrimination of all items (*a* = 2.46∼38.46), and the test information within the range of low-quality relationship was relatively high.

**Conclusion:**

The Chinese version of PDRQ-9 is a valid and reliable rating scale, which can measure the doctor-patient relationship among Chinese patients.

## 1. Introduction

For a long time, the patient-doctor relationship as perceived by the patient has been considered as a crucial concept in healthcare, for it can provide useful information in the prediction of patients’ adherence (1), safety (2) and treatment outcomes (3). However, at the beginning of this century, due to the market-oriented operation adopted in the healthcare industry (4), the doctor-patient relationship in China was often associated with mistrust or even conflict (5), while the safety of patients and caregivers in the hospital environment was also disrupted by amounts of external factors. In order to solve this problem, the government launched a new round of medical reform programs in 2009, aiming to realize the transformation of the medical industry from market-oriented operation to public welfare-oriented operation, as well as to establish a universal health coverage system in both rural and urban areas (6). With the continuous progress of medical reform, over 95% of the population in China is now covered by the basic medical insurance system, more than 1.3 billion people have access to affordable health services (7). Furthermore, the development of the hierarchical medical system and equalization of basic public health services also enable residents to obtain local medical resources in time (8). The optimization of macropolicies has gradually eliminated the unstable factors hindering patients’ and healthcare providers’ safety, while phenomena such as medicine misprescription and unaffordable health care costs, which used to cause conflicts between patients and doctors, have already become things of the past. In the present social environment, will the doctor-patient relationship in China undergo significant structural changes and provide more information for hospital safety management? To answer this question, it is urgent to introduce reliable measurement instruments for assessing doctor-patient relationship, as well as to explore its factor structures and psychometric properties, so as to provide a basis for reflecting the characteristics, structure and important related factors of today’s doctor-patient relationship in China.

In worldwide context, substantial efforts have been devoted to developing instruments for evaluating patient-doctor relationship. Among these instruments, the Patient-Doctor Relationship Questionnaire (PDRQ-9) (9) developed by Van der Feliz-Cornelis and her colleagues has been widely used to measure doctor-patient relationship from patients’ point of view. The PDRQ-9 was simplified from the PDRQ-15 scale, which originally consisted of two factors, respectively focusing on the empathic attitude of the doctor and medical symptoms of the patient. The final version of PDRQ-9 resulted in a preferably concise unidimensional scale containing the former factor. The PDRQ scale has been applied to assess doctor-patient relationship in various samples, including patients with physical diseases (10) and mental illness (11), as well has shown excellent reliability and validity in different cultural environments (11–13). Moreover, important findings of minor ratings in doctor-patient relationship among depressed compared to non-depressed participants were also revealed by research using PDRQ-9 (10, 13). Nevertheless, the psychometric properties and factor structures of PDRQ-9 have not been explored in Chinese culture environment yet. Besides, previous studies on PDRQ-9 have focused on the psychometric performance based on the classical test theory (CTT), but little attention has been paid to analyses using item response theory (IRT) methods. According to the situation stated above, this study aimed to evaluate the psychometric performance of PDRQ-9 among Chinese general hospital inpatients.

## 2. Materials and methods

### 2.1. Participants

The sample included 203 hospitalized patients recruited from August 2022 to October 2022 at the neurology, gastroenterology, obstetrics and gynecology ward of Peking Union Medical College Hospital, China. Inclusion criteria were to be inpatients aged 15 years or over, hospitalized for more than 24 h, and able to read and sign the informed consent form. The exclusion criteria included language barriers, limited writing skills, cognitive impairment/organic brain disorder/dementia, psychosis, and acute suicidal tendency. Every participant was informed of the study procedures, data collection and anonymization of all personal data, and electronic informed consent with valid electronic signatures was collected. Especially for participants under 18 years old, additional informed consent of a parent was required.

Uniformly trained psychiatrists or graduate students of psychiatry served as investigators. All participants were visited by investigators and informed about the investigation. After the informed consent was obtained, the participant would receive a QR code to scan, and then fill in the questionnaires using his or her own mobile phone. Investigators were available while the participants filled in the questionnaires, and offered help if any incomprehension occurs. A total of 203 questionnaires were collected while 2 invalid questionnaires were excluded for unidentifiable information provided. Investigators invited participants to complete retest questionnaire at the seventh day after the first questionnaire was completed. A total of 39 participants responded to the retest, with a response rate of 19.4%.

The Ethics Committee of Peking Union Medical College Hospital approved this study, with assurance that data would be reported in aggregate form anonymously.

### 2.2. Measurements

#### 2.2.1. Chinese version of the PDRQ-9

The Chinese version of PDRQ-9 is composed of 9 items, of which each item is rated on a 5-point Likert-type scale from 0 (not at all appropriate) to 4 (totally appropriate). Total scores are created by directly summing every item score. High total scores indicate better doctor-patient relationship as perceived by the patients (range 0–36). Previous studies have reported sufficient reliability of PDRQ-9 in other languages, with a 2-month test-retest correlation of 0.61 (6) and Cronbach’s alpha ranged from 0.94 to 0.97 (9, 14, 15).

The questionnaire was translated using a forward-backward translation method including steps of initial translation, synthesis of the translations, back translation, expert committee review, and test of the prefinal version (15, 16). Five psychiatrists first translated the questionnaire into the Chinese version from the English version and then synthesized into the initial version. Next, this version was back translated into English by a bilingual expert, and then compared with the original English version to identify any discrepancy in meaning. After the expert committee reached an agreement on the translated Chinese version, this version was used for pretest data collection. Nineteen patients recruited from the neurology ward took part in the pretest, and agreed that there was no discrepancy in meaning, thus forming the final version of the scale.

#### 2.2.2. Validation instruments

Referring to the research method used by Zenger and his colleagues to assess the validity of the German version of PDRQ-9 (13), our study introduced measurements of depressive symptoms to evaluate the convergent validity and divergent validity of the Chinese version scale. A significant correlation between ratings in the PDRQ-9 and depressive symptoms would support the convergent validity of the scale. In addition, the divergent validity of the PDRQ-9 will be assessed through correlations with theoretically unrelated constructs such as patients’ age (14).

This study evaluated patients’ depressive symptoms with the Patient Health Questionnaire Depression Scale-9 item (PHQ-9) (17) scale, which was developed according to the diagnostic criteria of major depressive disorders (MDD) following the Diagnostic and Statistical Manual of Mental Disorders, Fourth Edition (DSM-IV) (18). Participants were asked to rate perceived symptom burden during the past 2 weeks between 0 (not at all) and 3 (nearly every day), resulting in a total score ranging from 0 to 27. The reliability and validity of the Chinese version of PHQ-9 have been adequately validated in bountiful studies (19–21), with a generally accepted cut-off score of 10 (18, 19). The internal consistency of the PHQ-9 for this study was high (Cronbach’s α = 0.88).

#### 2.2.3. Sociodemographic questionnaire

Each participant’s information regarding age, gender, residence, family status, family income, level of education, and essential worker status was gathered through a demographic questionnaire.

### 2.3. Statistical analysis

The methods used to validate the Chinese version of PDRQ-9 were as follows, with a statistical significant criterion of *P* < 0.05:

(a) Descriptive statistics: Continuous variables and categorical variables were described in the form of mean ± standard deviation (mean ± SD) and numbers with percentages [n (%)] respectively. The Student’s *t* tests and one-way ANOVA tests were applied to compare the differences of PDRQ-9 scores among different groups.

(b) Item analysis: Corrected item-total correlations were calculated to measure the strength of the relationship between each item and the total score of the scale. A significant correlation coefficient larger than 0.4 is suggested as satisfactory (22).

(c) Structural validity: The sample was randomly split half to perform exploratory factor analysis (EFA) and confirmatory factor analysis (CFA), with IBM SPSS 20.0 and AMOS 27 respectively. Before the EFA was conducted, data suitability and sampling adequacy were checked using the Kaiser-Meyer-Olkin (KMO) value and Bartlett’s test of sphericity. During the principal components analysis, factors with an eigenvalue larger than 1 were extracted. A total factor loading of more than 60% was considered as acceptable (23). Secondly, a confirmatory factor analysis (CFA) [estimation method = diagonal weighted least square] was carried out. Acceptable model fit was defined by a root mean square residual (RMR) (24) value ≤ 0.05, a root-mean-square-error of approximation (RMSEA) (25) value ≤ 0.10, with comparative fit index (CFI) (26), normed fit index (NFI) (27), non-normed fit index (NNFI) (28), incremental fit index (IFI) (30), Tucker-Lewis index (TLI) (31) and goodness of fit index (GFI) (28) values ≥ 0.90 (29). Moreover, satisfactory model fit was defined by a RMSEA value below 0.05, with CFI and GFI above 0.90 (32).

(d) Convergent validity and divergent validity: The Pearson correlation coefficients between patients’ age, PHQ-9 rating, as well as total score of PDRQ-9 and its subscales were calculated. After the correlation analyses were conducted, all the participants were divided into two groups according to their ratings on PHQ-9. Patients who scored 10 or above were identified with significant depressive symptoms, while others were not. ANCOVA adjusted for age was conducted to verify whether there was statistical difference in PDRQ-9 ratings between participants with or without significant depressive symptoms. We hypothesized that the scores on PDRQ-9 would significantly correlate with PHQ-9 ratings but not patients’ ages, thus supporting the scale’s convergent validity and divergent validity.

(e) Reliability analysis: Cronbach’s α was used to evaluate the internal consistency of the Chinese version of PDRQ-9 and its subscales. The Pearson correlation coefficient between the first test and the retest was calculated to access the 7-day test-retest reliability. Cronbach’s α coefficients larger than 0.70 were considered as sufficient (33). Acceptable test-retest reliability was defined as statistical significance and Pearson correlation coefficient above 0.70 (34).

(f) IRT analysis: Since classical IRT analysis requires the construct of the scale to meet the unidimensional criterion (35), if the underlying construct of PDRQ-9 Chinese was found to contain more than one dimension in the factor analysis of step c), multi-dimension item response theory (MIRT) (36) analysis would be first carried out using the IRTPRO 6.0 software, with Samejima graded response model (37) adopted (38). The MIRT discrimination parameters (a) and intercept parameters (c) of each item would be computed according to the multidimensional model constructed in step (c), and the correlation θ as well as its 95% confidence interval between each two potential dimension would be calculated. If the upper limits of all confidence intervals were less than 1, it means that the potential dimensions do not completely overlap, and the data is consistent with multidimensional model rather than unidimensional model. Then on the basis of the analyses above, PDRQ-9 would be divided into subscales according to the multidimensional model. The unidimensionality assumption would be tested for each subscale using factor analyses. The IRT analysis with a fitted Semejima graded response model would be implemented for each subscale to estimate the discrimination parameters (a) and intercept parameters (c) of every item. At last, the information curves of each item and subscale would be drawn.

## 3. Results

### 3.1. Descriptive statistics

A total of 201 patients (46.58 ± 16.25 years) completed questionnaires in valid forms, of which 106 (52.7%) participants were female. The average PDRQ-9 score was 33.45 ± 4.44. The sociodemographic characteristics according to the PDRQ-9 scores of the patients were presented in Table 1. No significant difference in PDRQ-9 ratings among patients based on age, place of residence, educational levels, family status, family income or essential worker status was detected, but female patients scored significantly higher than male participants.

**TABLE 1:** Sociodemographic characteristics of total sample.

Characteristics	*N*	Proportion (%)	Mean	SD	*P*-value
Age					0.715^§^
15–20	12	6.0%	32.42	1.59	
21–40	64	31.8%	33.15	0.63	
41–60	79	39.3%	33.77	0.44	
>60	46	22.9%	33.52	0.64	
Gender					0.002**^†^
Male	95	47.3%	32.41	0.55	
Female	106	52.7%	34.39	0.31	
Residence					0.368^†^
City	153	76.1%	33.29	0.38	
Rural	48	23.9%	33.95	0.54	
Family status					0.646^§^
Single	32	15.9%	32.78	0.95	
Married	156	77.6%	33.50	0.35	
Divorced/Widowed	10	5.0%	34.80	0.80	
Other	3	1.5%	33.67	2.33	
Monthly family income					0.851^§^
Less than 4,000 RMB	29	14.4%	33.72	0.90	
4,000–8,000 RMB	81	40.3%	33.25	0.55	
More than 8,000 RMB	91	45.3%	33.55	0.41	
Essential worker status					0.844^§^
Employed/Student	93	46.3%	33.10	0.48	
Unemployed	33	16.4%	33.24	0.95	
Retired	59	29.4%	33.73	0.53	
Other	16	8.0%	34.94	0.42	
Education					0.853^§^
Elementary	56	27.9%	33.48	0.39	
College preparatory	43	21.4%	33.14	0.76	
University or higher	102	50.7%	33.64	0.65	

^†^Student’s *t*-test.

^§^One-way ANOVA test.

**P < 0.01.

### 3.2. Item analysis

To calculate corrected item-total correlation coefficients, Pearson correlation analysis was conducted between the score of each item in PDRQ-9 and the total score after subtracting the score of the item. The results showed that all the correlation coefficients ranged from 0.613 to 0.890 (Table 2), meeting the requirements of > 0.40 and showing statistical significance, indicating that all items had satisfactory consistency with the construct measured by the scale.

**TABLE 2:** Results of item analysis and factor loadings.

Item	Corrected item-total correlation	Loadings on factor 1	Loadings on factor 2
My PCP helps me	0.617***	0.895	0.082
My PCP has enough time for me	0.796***	0.856	0.318
I trust my PCP	0.831***	0.889	0.33
My PCP understands me	0.890***	0.843	0.423
My PCP is dedicated to helping me	0.888***	0.824	0.433
My PCP and I agree about the nature of my medical symptoms	0.613***	0.258	0.756
I can talk to my PCP	0.672***	0.174	0.871
I feel content with my PCP’s treatment	0.789***	0.291	0.832
I find my PCP easily accessible	0.743***	0.343	0.805

***P < 0.001.

### 3.3. Structural validity analysis

An exploratory factor analysis was conducted on a random half of the full sample (*n* = 101) to determine the number of factors. The KMO statistic was 0.879 and the significance of Bartlett’s test of sphericity [χ^2^(36) = 881.55, *P* < 0.001] indicated that the data was suitable for factor extraction. Principal component analysis employing the varimax rotation method was then implemented, and two common factors were extracted for eigenvalues above 1. These two factors accounted for 80.75% of the variation. The factor loading of each item was also shown in Table 2.

According to the meaning and factor loading of each item, items 1 to 5 could be classified into a dimension named **relationship quality**, which describes the trust and empathy experienced by patients during the treatment. Items 6 to 9 were classified as another dimension, named **treatment quality**, which describes patients’ satisfaction with the process and results of his or her treatment received. As Table 2 showed, the loading of each item on its corresponding factor was > 0.7.

**FIGURE 1:**
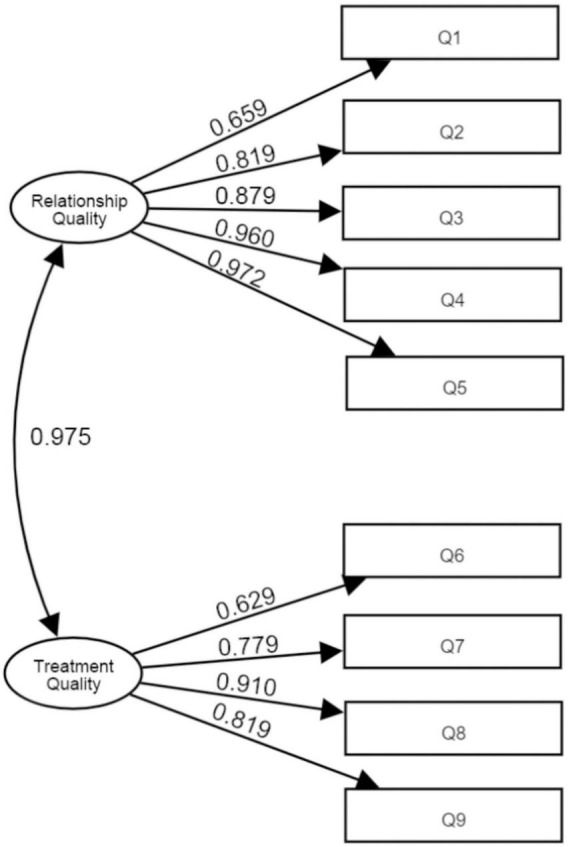
Factor structure of patient-doctor relationship questionnaire 9-item Chinese version.

Next, a confirmatory factor analysis with weighted least square estimation was conducted on the other random sample (*n* = 100) to test the two-factor model of relationship quality and treatment quality. The results showed that the factor loading of each item in the CFA model was > 0.6 (Figure 1), while all the model fit indexes were satisfactory (χ^2^/*df* = 1.494, GFI = 0.925, RMSEA = 0.071, RMR = 0.008, CFI = 0.985, NFI = 0.958, NNFI = 0.980, TLI = 0.980, IFI = 0.986), indicating excellent suitability of the two-factor model to the data.

### 3.4. Convergent and divergent validity analysis

Correlation analyses revealed significant negative correlation between scores on PHQ-9 and PDRQ-9 as well as its subscales, while no significant correlation was found between patients’ age and ratings of the scales above (Table 3).

**TABLE 3:** Descriptive statistics and correlation coefficients between variables.

Item	$\bar{x}$ ± *s*	PDRQ	PDRQ-RQ	PDRQ-TQ	PHQ-9	Age
PDRQ	33.45 ± 4.44	-				
PDRQ-RQ	18.66 ± 0.19	0.945***	-			
PDRQ-TQ	13.83 ± 0.14	0.901***	0.710***	-		
PHQ-9	6.32 ± 0.37	−0.264***	−0.196**	−0.309***	-	
Age	46.58 ± 16.25	0.046	0.034	0.054	−0.083	-

PDRQ, patient-doctor relationship questionnaire 9-item Chinese version; PDRQ-RQ, relationship quality subscale of PDRQ-9 Chinese version; PDRQ-TQ, treatment quality subscale of PDRQ-9 Chinese version; PHQ-9, patient health questionnaire depression scale-9 item; **P < 0.01; ***P < 0.001.

Patients were divided into two groups according to their scores on PHQ-9, those who scored 10 or above were identified with significant depressive symptoms while others were not. the mean score of participants with significant depressive symptoms (*N* = 47) was 32.15 ± 3.59, and that of participants without significant depressive symptoms (*N* = 54) was 33.91 ± 3.96. One-way ANCOVA controlling for age revealed significantly higher mean scores on PDRQ-9 (*F* = 5.56, *P* = 0.019) in the group with significant depressive symptoms with a small effect size (partial η^2^ = 0.028), while the partial η^2^ of age was less than 0.001 (*F* = 0.09, *P* = 0.764). This result demonstrates acceptable convergent validity.

### 3.5. Reliability analysis

Cronbach’s α coefficients of the PDRQ-9 full scale, relationship quality and treatment quality subscale were 0.933, 0.932, and 0.865, respectively. For the full scale, the unequal-length Spearman-Brown split-half reliability was 0.839, while the 7-day test-retest reliability (*r*) was 0.730 (*P* < 0.001). These above analyses indicated good reliability of the scale.

### 3.6. Analysis based on item response theory

Since two underlying dimensions of PDRQ-9 Chinese were revealed in the factor analyses, MIRT analysis was first carried out. Samejima graded response model was adopted to estimate discrimination parameters (a) and intercept parameters (c) for each item in the full scale. The discrimination parameters estimated ranged from 2.83 to 28.43 (Table 4), which were all above 1.70 and considered very high (36). The MIRT model suggested a correlation (θ) of 0.93 between the two dimensions, with a 95% confidence interval of [0.89, 0.97], of which the upper limit was less than 1, indicating that the two dimensions were highly correlated but did not completely overlap, and the constructs measured by the Chinese version of PDRQ-9 were consistent with two-factor model rather than unidimensional model.

**TABLE 4:** Item content of patient-doctor-relationship questionnaire (PDRQ-9) full scale and multidimensional item response theory (MIRT) item parameter estimates.

Items on the PDRQ-9	a1	a2	c1	c2	c3	c4
My PCP helps me	3.35	-	6.06	5.38	4.32	0.88
My PCP has enough time for me	4.92	-	9.90	7.20	6.10	0.19
I trust my PCP	9.09	-	16.79	15.00	13.20	2.51
My PCP understands me	28.43	-	50.21	44.15	29.13	−0.10
My PCP is dedicated to helping me	27.45	-	48.77	28.84	1.93	-
My PCP and I agree about the nature of my medical symptoms	-	2.82	6.49	4.97	3.27	0.02
I can talk to my PCP	-	3.87	7.97	4.82	1.23	-
I feel content with my PCP’s treatment	-	7.33	12.92	11.98	7.75	1.46
I find my PCP easily accessible	-	6.11	9.61	8.53	2.09	-

**FIGURE 2:**
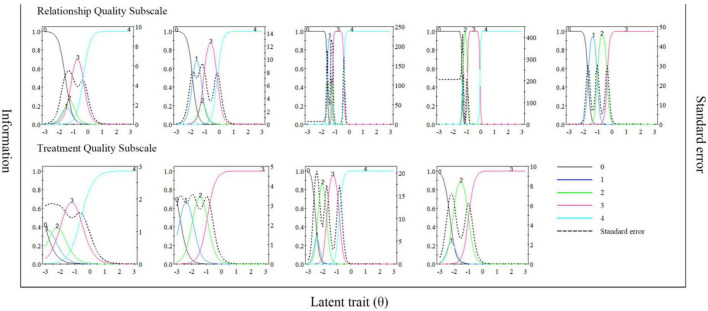
Item characteristic curves and item information curves of items in the PDRQ-9.

**FIGURE 3:**
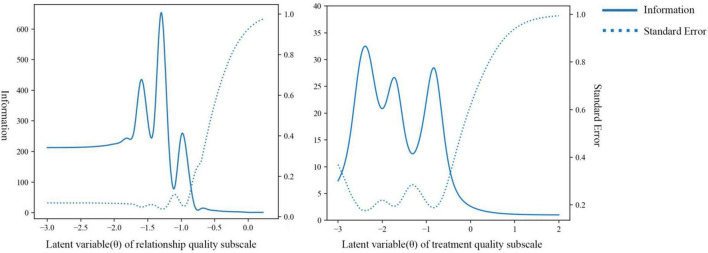
Item information curves of PDRQ-9 subscales.

Principal component analysis was then implemented to examine the unidimensionality assumption for each subscale. For the relationship quality subscale, the eigenvalue of the first factor was 4.035 accounting for 80.96% of the variation, and the second eigenvalue was 0.477, which was less than one-third of the first eigenvalue. For the treatment quality subscale, the eigenvalue of the first factor was 2.897 accounting for 80.96% of the variation, and the second eigenvalue was 0.526, which was also less than one-third of the first eigenvalue. These results showed that both subscales met the unidimensionality assumption, thus suitable for IRT analysis. The discrimination parameters estimated ranged from 2.46 to 38.46 (Table 5), which were all considered very high (36). The category characteristics curves and item information curves for all items were shown in Figure 2, while the item information curves of two subscales were presented in Figure 3. From the information curves, it can be seen that the test information within the range of lower latent variable level was relatively high, suggesting that this scale is more suitable for distinguishing and screening doctor-patient relationship with more difficulty or conflicts.

**TABLE 5:** Item content of patient-doctor-relationship questionnaire (PDRQ-9) subscales and item response theory (IRT) item parameter estimates.

Subscale	Items on the PDRQ-9	a1	c1	c2	c3	c4
Relationship quality	My PCP helps me	4.20	6.59	5.87	4.79	1.33
	My PCP has enough time for me	5.63	10.52	7.14	6.18	0.78
	I trust my PCP	28.59	45.17	37.21	34.59	10.59
	My PCP understands me	38.46	51.61	49.38	36.86	1.90
	My PCP is dedicated to helping me	11.15	18.60	11.41	3.72	-
Treatment quality	My PCP and I agree about the nature of my medical symptoms	2.46	7.78	6.26	4.61	1.42
	I can talk to my PCP	3.67	10.28	7.06	3.42	-
	I feel content with my PCP’s treatment	8.32	20.69	19.47	14.21	6.79
	I find my PCP easily accessible	4.98	11.40	10.43	4.90	-

## 4. Discussion

In this study, inpatients from a general hospital in China were recruited as participants to explore the reliability, validity and psychometric characteristics of PDRQ-9 for measuring doctor-patient relationship in Chinese culture environment. The results showed that the Chinese version of PDRQ-9 was consistent with the two-factor model of relationship quality and treatment quality, showing satisfactory internal consistency, discrimination, reliability and validity, and can obtain more adequate information when identifying and screening doctor-patient relationship with more difficulty or conflicts, providing a feasible choice for evaluating doctor-patient relationship in Chinese culture. Besides suggesting supporting evidence for the cross-cultural reliability and validity of PDRQ-9, this study also revealed some phenomena that had not been observed in previous studies:

Firstly, different from the unidimensional structure found in other language versions of PDRQ-9, factor analyses on the Chinese version suggested the two-factor model of relationship quality and treatment quality, with the former factor reflecting the empathy and trust experienced by the patient during the treatment, while the latter factor representing patients’ satisfaction with the process and results of his or her treatment received. Such difference in dimensional structure could be explained by the cultural differences between China and western countries. Amounts of previous studies have already revealed differences in attribution styles of people from collectivist culture and individualistic culture (39): Participants grew up in collectivist cultural environments are often sensitive to environmental factors when attributing, and tend to make more contextual references instead of dispositional references, while people who grew up in individualistic cultural environments tend to ignore the background and attribute others’ behavior to their own traits. The above phenomena provided a potential explanation for the dimensional differences of PDRQ-9 in different cultural environments: Chinese patients would probably take more environmental factors into consideration when attributing doctors’ behavior. For example, they might believe that whether they were satisfied with the treatment they received not only depended on their doctors’ ability or conscientiousness, but was also limited by the conditions provided by the hospital, thus leading to changes in the measured construct of the scale. The phenomenon that difference in cultural environments leads to changes in factor constructs of scales has also been observed in previous studies on cross-cultural adaptation of various psychological measurement instruments such as the Big Five Personality Scale (40) and the Indecisiveness Scale (41), suggesting the importance of carefully evaluating factor structures before applying scales or questionnaires across different cultures. However, it is still worth noting that in Bangladesh, which is also generally considered to be a typical collectivist cultural country (42), the PDRQ-9 shows the same unidimensional structure as in western countries (43), which probably suggests that the difference in dimensional structure may not be fully explained by the individualism-collectivism tendency of culture. In order to further explore the influence of cultural environment on doctor-patient relationship assessment, subsequent studies may supplement the assessment of collectivism, individualism (44) and attribution style (45) while measuring doctor-patient relationship, and explore the influence of cultural psychological variables on the model fit indexes of PDRQ-9, so as to provide further empirical evidence for the explanation above.

Secondly, this study applied statistical methods under the framework of item response theory for the first time to evaluate the psychometric performance of PDRQ-9. The results indicated that each item showed satisfactory performance in discrimination in both MIRT model of the full scale and unidimensional IRT model of two subscales. The item information curves suggested that PDRQ-9 could provide more adequate information in identifying patients experiencing more difficult doctor-patient relationship and higher risk of conflict. In conclusion, PDRQ-9 is more suitable for screening and risk assessment of difficult doctor-patient relationship, and can provide sensitive indicators for intervention studies on such relationship, but its performance in measuring good doctor-patient relationship is relatively ordinary. Future studies may further examine whether such information function pattern is consistent in other cultural environments, and explore ways to improve the performance of this scale to measure good doctor-patient relationship.

In addition to the new phenomena observed above, this study could also provide guidance for clinical work: Consistent with previous findings by Zenger and his colleagues (13), this study again confirmed the association between the quality of the doctor-patient relationship as measured by PDRQ-9 and the level of patients’ depressive symptoms. Although the cross-al study method adopted in our study could not elucidate the causal relationship between depression and doctor-patient relationship, it still suggested clinicians to pay more attention to the maintenance of doctor-patient relationship when treating patients with significant depressive symptoms, and also be alert to the risk of depression among patients experiencing doctor-patient conflicts. Future studies can explore the causal relationship between depression and doctor-patient relationship through cross-lag design (46), thus providing specific guidance for health maintenance strategies in clinical practice.

This study also had some limitations. First, as there is still a lack of instruments for assessing doctor-patient relationship as perceived by the doctor and patients’ treatment satisfaction that has been strictly validated and widely used in Chinese culture environment, this study only introduced PHQ-9 questionnaire, of which the Chinese version has been validated in previous studies, to measure depressive symptoms as a criterion, leading to the use of only a single measure to assess the convergent validity of PDRQ-9. Future studies should include other measures to carry out a better evaluation of convergent validity and discriminant validity. Secondly, participants of this study were all inpatients recruited from ward of physical diseases in a general hospital, which might result in a certain degree of selection bias. In the future, the measurement of the scale in various samples such as patients from psychiatric hospitals, outpatients and community clinics should be supplemented to further verify the robustness of its psychometric characteristics.

## Data availability statement

The raw data supporting the conclusions of this article will be made available by the authors, without undue reservation.

## Ethics statement

The studies involving human participants were reviewed and approved by the Ethics Committee of Peking Union Medical College Hospital. All patients/participants provided their electronic informed consent to participate in this study. For participants under 18, electronic informed consent to participate in this study was provided by the participants’ legal guardian/next of kin.

## Author contributions

YW drafted the manuscript. YW and AW contributed to the data analysis, results, and finalized the manuscript. YJ, LS, and JW proposed the concept and design. YW, AW, YD, WG, LW, JL, JD, JH, and JJ made important contributions to data collection. All authors read and approved the final manuscript.
