# Supplementary material for: The Chinese version of patient-doctor-relationship questionnaire (PDRQ-9): Factor structure, validation, and IRT psychometric analysis
Source: Front Psychiatry. 2023 Feb 16;14:1117174. doi: 10.3389/fpsyt.2023.1117174 (PMC9978500; doi:10.3389/fpsyt.2023.1117174)
Supplement: Supplementary file 1 [file Table_1.docx]

Supplementary Material

# Supplementary Tables

**Supplementary Table 1. Item contents of the Chinese version of PDRQ-9**

| **Item content** | **Not at all appropriate** | **Somewhat appropriate** | **Appropriate** | **Mostly appropriate** | **Totally appropriate** |
| --- | --- | --- | --- | --- | --- |
| My doctor helps me |  |  |  |  |  |
| My doctor has enough time for me |  |  |  |  |  |
| I trust my doctor |  |  |  |  |  |
| My doctor understands me |  |  |  |  |  |
| My doctor is dedicated to helping me |  |  |  |  |  |
| My doctor and I agree about the nature of my medical symptoms |  |  |  |  |  |
| I can talk to my doctor |  |  |  |  |  |
| I feel content with my doctor’s treatment |  |  |  |  |  |
| I find my doctor easily accessible |  |  |  |  |  |

Instructions: In the following section, you will read nine statements that a person can make about his/her doctor. Please choose the appropriateness of each statement for your doctor.
